# Coeliac disease detection with an IgA/IgG-deamidated gliadin peptide based point of care test in community pharmacies

**DOI:** 10.1007/s11096-019-00793-8

**Published:** 2019-02-16

**Authors:** Michelle S. Lau, David S. Sanders

**Affiliations:** Academic Department of Gastroenterology, Room P18, Royal Hallamshire Hospital, Sheffield Teaching Hospitals, Sheffield, S10 2JF UK

**Keywords:** Case detection, Coeliac disease, Community pharmacy, Deaminated gliadin peptide, DGP, Point of care test, Primary care, Simtomax^®^

## Abstract

*Background* Coeliac disease affects 1% of the population, but 75% remain undiagnosed. *Objective* To conduct a case finding feasibility and efficacy study for the detection of coeliac disease in community pharmacies. *Setting* Six community pharmacies across Sheffield, UK. *Method* A prospective study was performed using a point of care test, Simtomax^®^ (IgA/IgG-deamidated gliadin peptide) (C-test) in pharmacies. Pharmacy customers with symptoms suggestive of or risk factors for coeliac disease were tested with the C-test. Positive individuals were referred for a gastroscopy with duodenal biopsies alongside conventional serology. People with known coeliac disease, those on a gluten free diet or those who were investigated for coeliac disease were excluded. *Main outcome measure* The case detection rate and the uptake rate of the C-test and gastroscopies. *Results* Five-hundred participants fulfilled the inclusion criteria and were tested with the C-test (369 females, 73.8%; age range 18–87, median 49). The C-test uptake rate was 63%, and the positive rate was 7.2% (36/500). Twenty-seven positive participants (75%) underwent further investigations, confirming three new cases of coeliac disease (0.6%). *Conclusion* It was feasible to use the C-test as a case finding tool in pharmacies. There was good uptake for the C-test, although the case detection rate and the test specificity were low. Based on this, the C-test has a limited role in case finding in a community pharmacy setting.

## Impacts on practice

- The use of the C-test as a case finding tool in community pharmacies in the UK is practically feasible with a good uptake rate from pharmacy customers.
- The case detection rate of the C-test in practice is relatively low at 0.6%. Additionally, the false positive rate is high at 89.9%, meaning that approximately only 1 in 10 individuals with a positive C-test will have have coeliac disease after further investigations.
- Case finding with the use of the C-test in the community pharmacy setting is not recommended, in view of the properties of the test.

## Introduction

Coeliac disease is a systemic autoimmune disease associated with gastrointestinal and extra-gastrointestinal symptoms, triggered by gluten in genetically susceptible individuals affecting approximately 1% of the general population worldwide based on population screening studies [1–3]. One of the major challenges with coeliac disease is that 75% of patients remain undiagnosed [4] despite national guidelines for coeliac testing [5]. We have previously reported that one-third of patients were seen by other medical or surgical specialties with coeliac disease related symptoms before being diagnosed, with a mean delay in diagnosis of 4.9 years [6].

There has been an increased recognition of the changing presentation of coeliac disease in the past two decades, from the classical symptoms of diarrhoea and weight loss to the more commonly seen non-classical features such as fatigue and anaemia [6–10]. These symptoms can be subtle and difficult to recognise as a presenting symptom of coeliac disease.

Thus there has been a drive to screen for coeliac disease in at risk individuals in primary care. Previous primary care case finding studies have shown improved case detection rates through serological screening in high risk patients, with a coeliac disease detection rate of approximately 2–3% [11–13]. A recent proof of concept study demonstrated that screening 551 high risk individuals with a point of care test in community pharmacies led to a positive test in 9.4% of the participants. The study also demonstrated high levels of satisfaction from the pharmacists and the participants with the service, suggesting feasibility of running such a service by allied health care professionals in primary care. However, there was no data pertaining to the subsequent follow up or biopsy results to confirm the number of coeliac disease cases detected [14].

Despite national serological screening guidelines, it appears that clinicians do not uniformly follow this practice. This was demonstrated in our multicentre study in the UK, where only 30% of anaemic patients had serology performed prior to their gastroscopies [15]. This result mirrors that of a US study where only 30% of patients suspected of coeliac disease had serology performed [16]. Moreover, under the current climate of the National Health Service, increasingly longer waits to access primary care are not uncommon. Further delay is encountered with the arrangement of a blood test for coeliac serology and then obtaining the results and onward referral for duodenal biopsies. All these issues suggest insufficiencies in our current case finding strategy.

This necessitates an alternative approach to improve case detection. Community pharmacies could potentially provide a unique opportunity to recognise undiagnosed coeliac disease in primary care with the help of community pharmacists. It has previously been shown the deamidated gliadin peptide (DGP) based point of care test, Simtomax^®1^ (C-test), had comparable diagnostic performance to conventional serology (IgA-endomysial [EMA] and IgA-tissue transglutaminase [TTG] antibodies) [17]. This finger prick point of care test offers an additional advantage of rapid result availability within 10 minutes, which makes it ideal to be used in a community setting.

## Aims of the study

We aimed to evaluate the role of the C-test for the detection of coeliac disease in at risk or symptomatic individuals in a primary care community setting. The primary outcome measure was the coeliac disease detection rate. Secondary outcomes included the uptake rate of the C-test and subsequent gastroscopy with duodenal biopsies.

## Ethics approval

The study protocol was approved by the Yorkshire and the Humber Research Ethics committee and registered with the local research and development department of Sheffield Teaching Hospital NHS Foundation Trust under the registration number STH19172. Written consent was obtained from all individual participants included in the study.

## Methods

### Study design and recruitment

A prospective case finding study was conducted in six community pharmacies across the city of Sheffield (Darnall, Foxhill, Manor Top, Wicker, and Barnsley Road) over a 4-month period. Customers entering the pharmacies with symptoms or risk factors for coeliac disease indicated for coeliac screening by the National Institute of Clinical Excellence (NICE) were approached (see Table 1). Customers obtaining relevant medications either through prescription or over the counter were approached by the pharmacists and referred to the researcher for study participation. The relevant medications included treatment for possible symptoms of coeliac disease (e.g. anti-spasmodics for irritable bowel) or conditions that may be associated with coeliac disease (e.g. insulin for type 1 diabetes). These target medications are listed in Table 2. Posters advertising for the study were also in place in all pharmacies which customers could enquire further for study participation. Inclusion criteria included individuals aged 18 or over, with any symptoms or risk factors listed in Table 1, and/or taking medications listed in Table 2. Exclusion criteria included people with known coeliac disease, those on a gluten free diet, those who had previous or current investigations for coeliac disease, and pregnancy.

**Table 1:** Symptoms of and risk factors for coeliac disease that fulfil the inclusion criteria for the study based on the National Institute of Clinical Excellence guidelines

Persistent unexplained abdominal or gastrointestinal symptoms	First degree relatives of people with coeliac disease
Irritable bowel syndrome	Metabolic bone disorders (reduced bone mineral density/osteomalacia/osteoporosis)
Prolonged fatigue/tired all the time	Unexplained neurological symptoms (peripheral neuropathy or ataxia)
Unintentional weight loss	Unexplained subfertility or recurrent miscarriage
Severe or persistent mouth ulcers	Persistently raised liver enzymes with unknown cause
Iron, vitamin B12 or folate deficiency	Dental enamel defects
Type 1 diabetes	
Autoimmune thyroid disease

**Table 2:** Types of medications taken by an individual that would trigger recruitment into the study

Drug group	Example drugs
Antispasmodics	Mebeverine, buscopan, spasmonal
Anti-reflux medications	Proton pump inhibitor, gaviscon, ranitidine
Anti-diarrhoeal	Loperamide
Laxatives	Senna, movicol, sodium docusate
Anti-emetic	Domperidone, metoclopramide
Supplements	Ferrous sulphate, vitamin B12, folate
Thyroid medications	Thyroxine, carbimazole
Insulin	Insulin (for type 1 diabetics)

Individuals who met the eligibility criteria were approached by the researcher and consented for the study. The C-test was performed in a private consultation room within the pharmacies. The C-test results were obtained within 10 minutes and participants were informed of the results in real time. Those with a positive C-test were referred for a gastroscopy with duodenal biopsies and conventional serology at the Royal Hallamshire Hospital, Sheffield. Those with a negative C-test were advised to see their general practitioner if necessary or if their symptoms persisted.

### Point of care test, Simtomax^®^

Simtomax^®^ is a point of care test for coeliac disease (C-test) manufactured by Augurix Diagnostics, Rheinfelden, Switzerland. It detects both IgA- and IgG-deamidated gliadin peptide (DGP), as well as the presence of IgA. The assay is based on lateral flow immunochromatography using colloidal gold antihuman antibodies as a signal detector. A sample of 25 μl of capillary venous blood was obtained through a simple finger prick technique. The blood sample was then applied to the test device, followed by the application of five drops of the provided buffer solution. The result was available after 10 min. Positive results were indicated by the presence of a solid red test line for IgA-DGP and/or IgG-DGP positivity. A second single red line indicated the presence of IgA. An in-built red control line ensured a correctly functioning test.

### Coeliac serology

IgA-tissue transglutaminase (TTG) antibodies were assayed using enzyme-linked immunosorbent assay (ELISA) kits (Aesku Diagnostics, Wendelsheim, Germany). A TTG titre of > 7 U/ml was regarded as positive as per the manufacturer’s guidance. IgA-EMA was detected by immunofluorescence on primate oesophagus sections (Binding Site, Birmingham, UK). Total IgA was measured on a Behring BN2 nephelometer (Haywards Heath, West Sussex, UK).

### Histological evaluation

In total, at least five biopsies were taken from the duodenum with a single bite per pass technique, including at least one biopsy from the duodenal bulb and four quadrantic biopsies from the second part of the duodenum. Each biopsy was fixed in formalin at the time of the gastroscopy. Specimens were then processed, orientated and embedded in paraffin wax by the pathology department. Standard 3 μm thick sections at three levels were stained with haematoxylin and eosin, and reported by gastrointestinal histopathologists without knowledge of the C-test or serology results. Villous atrophy was graded according to the modified Marsh criteria.

### Definitions of diagnoses

The definition of coeliac disease was based on positive serology (positive TTG and/or EMA) with Marsh 3 histology. Potential coeliac disease was defined as positive serology with no villous atrophy (Marsh 0–2). Supporting information such as human leucocyte antigen genotype, family history and response to a gluten free diet were taken into account.

### Statistics

Data were summarised by descriptive statistics, including counts and percentages for categorical data, and medians and ranges for continuous parameters. The statistics department of the University of Sheffield was consulted for the sample size. We anticipated a 3% case detection rate in our cohort based on previous primary care case finding studies. Using an exact binomial test, a sample size of 500 would have 88% power to detect the difference between the estimated 3% prevalence in the study cohort and the estimated 1% prevalence of coeliac disease in the general population.

## Results

Eight hundred and two pharmacy customers were approached for the study. Eight individuals (1.1%) who were approached already had a coeliac disease diagnosis and were therefore excluded. Five hundred individuals met the eligibility criteria and agreed to undertake the C-test at the pharmacies and further investigations if they were tested positive, giving a C-test uptake rate of 63% (500/794). There were 369 females (73.8%), and the age range was 18-87 (median age 49). Thirty-six participants (36/500 = 7.2%) were tested positive, of which 27 (75%) subsequently underwent further evaluation with conventional serology and a gastroscopy with duodenal biopsies. Of the remaining nine positive participants who did not have further investigations, seven participants changed their mind at the pharmacy and declined further investigations, one participant wanted time to consider further tests and eventually declined a gastroscopy, and one agreed to further tests but did not attend for the gastroscopy appointment and subsequently declined further tests over follow up phone call. Two participants were diagnosed with coeliac disease based on positive serology and Marsh 3 villous atrophy, and one was diagnosed with potential coeliac disease based on gluten related symptoms and positive serology without villous atrophy. The patient with potential coeliac disease was commenced on a gluten free diet in view of her gluten related symptoms. Excluding the nine patients who did not have gastroscopies to confirm the diagnosis, the coeliac disease prevalence was 0.6% (3/491) including the latter patient with potential coeliac disease. Table 3 illustrates the participants’ presenting characteristics.

**Table 3:** Participants presenting characteristics

Presenting feature	Number of participants	%
Persistent unexplained abdominal or gastrointestinal symptoms	441	88.2
Irritable bowel syndrome	176	35.2
Prolonged fatigue	261	52.2
Unintentional weight loss	48	9.6
Severe or persistent mouth ulcers	68	13.6
Iron, vitamin B12 or folate deficiency	47	9.4
Type 1 diabetes	4	0.8
Autoimmune thyroid disease	47	9.4
First degree relatives of people with coeliac disease	21	4.2
Metabolic bone disorders (reduced bone mineral density/osteomalacia/osteoporosis)	20	4
Unexplained neurological symptoms (peripheral neuropathy or ataxia)	1	0.2
Unexplained subfertility or recurrent miscarriage	9	1.8
Persistently raised liver enzymes with unknown cause	2	0.4

## Discussion

This is the first study to demonstrate both the feasibility and efficacy of using the C-test in community pharmacies for the detection of coeliac disease. Allied health care professionals are an asset for supporting and relieving the workload of clinicians. The aim of offering the C-test to at risk individuals at community pharmacies was to increase the detection of coeliac disease and reduce delays in diagnosis as an alternative approach in primary care.

In this study we have shown that there was a good response from pharmacy customers in regards to the uptake rate of the C-test and subsequent further investigations as required. The positive C-test rate was 7.2%, which was similar to the 9.4% positive rate from the previous proof of concept study using the C-test in pharmacies by Urwin et al. [14], although no subsequent investigations were undertaken to confirm the diagnosis in that study.

The coeliac disease detection rate of 0.6% in our study was however much lower than anticipated. There are several possible reasons for this. One of the reasons could be because the existing caseload of coeliac disease is higher than average due to the presence of our tertiary referral centre for coeliac disease in Sheffield, and the heightened awareness for active coeliac testing by local general practitioners because of that. During the recruitment period, eight pharmacy customers approached for the study already had a known diagnosis of coeliac disease, giving a 1.1% known coeliac disease prevalence among the 802 individuals approached, which is higher than the UK average detected prevalence [4]. Conducting the same study in other areas may be useful in determining the true case finding potential of community point of care testing. Another potential factor which may have contributed to the low detection rate is that 25% of participants who tested positive did not proceed to further investigations to confirm or exclude coeliac disease. Therefore, it is possible that undiagnosed cases have not been accounted for. Lastly, most recruited individuals were passively approached to be offered the C-test, as opposed to the individual actively seeking for medical advice for their symptoms. This may have led to the inclusion of individuals with mild or insignificant symptoms which they would not have sought for medical help in the first instance. It is conceivable that the case detection rate may possibly be higher if the C-test was performed in individuals with symptoms significant enough for them to actively request for coeliac testing at the pharmacies.

Although this study was not designed to assess the diagnostic accuracy of the C-test, it was observed that the false positive rate was very high when used in the pharmacy setting. Of the 27 customers with a positive C-test who all underwent further diagnostic testing, only three were found to have coeliac disease, giving a false positive rate of 88.9%. Based on the low specificity of this test when used in the community, the C-test cannot be recommended to be used as a case finding tool in the pharmacy setting due to the high number of positive individuals who would be subjected to unnecessary further investigations.

## Conclusion

We demonstrated the feasibility of using the C-test for the detection of coeliac disease in the primary care sector in community pharmacies. Although there was good uptake for undertaking the C-test among pharmacy customers, the coeliac disease yield was much lower than anticipated. The C-test did not increase case detection as estimated, therefore it may have a limited role as a case finding tool in a community pharmacy setting.
